# Supplementary material for: Modulation of Paracellular-like Drug Transport across an Artificial Biomimetic Barrier by Osmotic Stress-Induced Liposome Shrinking
Source: Pharmaceutics. 2022 Mar 28;14(4):721. doi: 10.3390/pharmaceutics14040721 (PMC9027509; doi:10.3390/pharmaceutics14040721)
Supplement: Supplementary file 1 [file pharmaceutics-14-00721-s001.zip › pharmaceutics-1638986-supplementary.pdf]

Calcein

Osmolality	50				
Barrier	PermeaPad				
Time	A	B	C	mean	SD
1800	2.352798	2.419319	2.384228	2.385448	0.027171
2700	3.966294	3.967779	3.770734	3.901603	0.09254
3600	5.539146	5.589198	5.150847	5.426397	0.195912
4500	6.917449	7.364132	6.651946	6.977842	0.293868
5400	8.612041	9.296521	8.337431	8.748665	0.403289
Flux (µg/cn	0.001719	0.001906	0.001643	0.001756	0.00011
Papp (10-6	0.592707	0.657117	0.566575	0.605466	0.038049
Mass in do	27134.89	27134.89	27134.89	27134.89	0
Mass in do	0	25648.6	27462.69	17703.76	12540.34
Mass Balar	N/A	94.55686	101.2388	97.89781	3.340949

Osmolality	150				
Barrier	PermeaPad				
Time	A	B	C	mean	SD
1800	2.722269	2.992343	2.097506	2.604039	0.374759
2700	3.937933	4.178911	3.132366	3.749736	0.447495
3600	6.274996	5.740072	4.447429	5.487499	0.767179
4500	8.090355	7.559222	5.641531	7.097036	1.051791
5400	10.37092	9.144638	6.948258	8.821273	1.415882
Flux (µg/cn	0.002161	0.001743	0.001357	0.001754	0.000328
Papp (10-6	0.7452	0.600954	0.467842	0.604665	0.113262
Mass in do	26354.29	26354.29	26354.29	26354.29	3.64E-12
Mass in do	0	30727.65	36570.53	22432.73	16040.68
Mass Balar	N/A	116.6292	138.7914	127.7103	11.08109

Osmolality	250				
Barrier	PermeaPad				
Time	A	B	C	mean	SD
1800	1.025334	0.83875	1.316374	1.060152	0.196538
2700	1.737059	1.37486	2.173228	1.761716	0.326399
3600	2.435813	1.958336	3.097578	2.497242	0.467118
4500	3.578397	2.754101	3.841598	3.391365	0.463248
5400	4.419303	3.455611	4.897316	4.25741	0.599603
Flux (µg/cn	0.000959	0.000735	0.000981	0.000892	0.000111
Papp (10-6	0.330624	0.25337	0.338324	0.307439	0.038362
Mass in do	23200	23200	23200	23200	0
Mass in do	0	25810.18	28863.48	18224.55	12946.85
Mass Balar	N/A	111.2657	124.4327	117.8492	6.583503

Osmolality	300				
Barrier	PermeaPad				
Time	A	B	C	mean	SD
1800	0.786753	1.377624	1.092924	1.085767	0.241275
2700	1.510291	2.111147	1.907748	1.843062	0.249526
3600	2.278859	2.490094	3.060344	2.609766	0.330071
4500	3.256127	3.977803	4.009698	3.747876	0.347963

The time is represented in seconds

The green cells represent the cumulative amount (µg) of drug permeated at each timepoint for the replicates

If not other stated the membrane has been pretreated for 30minutes

5400	4.294801	5.137641	4.688903	4.707115	0.344329
Flux (µg/cn	0.000974	0.001043	0.001033	0.001016	3.06E-05
Papp (10-6	0.335706	0.359643	0.356088	0.350479	0.010546
Mass in do	26354.29	26354.29	26354.29	26354.29	3.64E-12
Mass in do	0	23377.63	28425.53	17267.72	12382.81
Mass Balar	N/A	88.70523	107.8592	98.28223	9.577

Osmolality	350				
Barrier	PermeaPad				
Time	A	B	C	mean	SD
1800	1.018616	1.059631	0.845601	0.974616	0.092751
2700	1.980759	1.783252	1.556532	1.773515	0.173327
3600	2.930949	2.499138	2.490306	2.640131	0.205671
4500	4.936015	3.256273	3.165155	3.785814	0.814165
5400	5.874554	4.006648	4.246987	4.709396	0.829713
Flux (µg/cn	0.001407	0.000819	0.000935	0.001054	0.000255
Papp (10-6	0.485331	0.282263	0.322276	0.36329	0.087829
Mass in do	26354.29	26354.29	26354.29	26354.29	3.64E-12
Mass in do	0	30422.98	29677.51	20033.5	14169.09
Mass Balar	N/A	115.4537	112.6259	114.0398	1.413877

Osmolality	450				
Barrier	PermeaPad				
Time	A	B	C	mean	SD
1800	1.557624	1.616357	0.707384	1.293788	0.415343
2700	2.67906	2.555837	1.158846	2.131248	0.68943
3600	4.200732	3.601665	1.948268	3.250222	0.952552
4500	5.70179	4.67077	2.517171	4.296577	1.326767
5400	7.219363	5.606752	3.013449	5.279854	1.732546
Flux (µg/cn	0.001594	0.001122	0.000663	0.001126	0.00038
Papp (10-6	0.549663	0.386809	0.228753	0.388409	0.131016
Mass in do	26354.29	26354.29	26354.29	26354.29	3.64E-12
Mass in do	0	28456.95	23846.05	17434.33	12470.82
Mass Balar	N/A	107.9997	90.49406	99.24689	8.752829

Osmolality	900				
Barrier	PermeaPad				
Time	A	B	C	mean	SD
1800	1.775204	1.743988	2.503923	2.007705	0.351111
2700	2.971425	2.974116	4.38893	3.444823	0.667585
3600	4.260474	4.150725	6.045805	4.819001	0.868637
4500	5.5816	6.078119	7.130985	6.263568	0.645983
5400	7.150297	7.665502	8.919503	7.911767	0.74297
Flux (µg/cn	0.001484	0.001661	0.00173	0.001625	0.000103
Papp (10-6	0.511891	0.572683	0.596675	0.560416	0.035683
Mass in do	23200	23200	23200	23200	0
Mass in do	0	27521.53	27501.44	18340.99	12969.04
Mass Balar	N/A	118.6603	118.5791	118.6197	0.040601

Osmolality	50

Barrier	Support sheet				
Time	A	B	C	mean	SD
1800	6.53103	5.438897	5.058651	5.676193	0.624076
2700	14.86624	14.29101	14.65038	14.60254	0.23726
3600	27.16886	21.26934	16.06483	21.50101	4.536158
4500	25.20685	27.66729	22.69408	25.18941	2.030342
5400	33.02143	36.77013	29.5732	33.12159	2.938989
Flux (µg/cn	0.007036	0.008449	0.006341	0.007275	0.000877
Papp (10-6	2.426108	2.913362	2.186697	2.508722	0.302357
Mass in do	23200	23200	23200	23200	0
Mass in do	17651.84	17651.84	18439.77	17914.48	371.4372
Mass Balar	76.22783	76.24399	79.60925	77.36036	1.590221

Osmolality	300				
Barrier	Support sheet				
Time	A	B	C	mean	SD
1800	3.277501	5.641885	6.085028	5.001471	1.232382
2700	9.869295	15.35484	10.74758	11.99057	2.405766
3600	10.82345	16.8339	11.76148	13.13961	2.640178
4500	21.70973	28.57673	24.48867	24.92504	2.820373
5400	25.02083	43.88759	37.70102	35.53648	7.852919
Flux (µg/cn	0.006147	0.009968	0.008553	0.008223	0.001577
Papp (10-6	2.119812	3.437291	2.94916	2.835421	0.543838
Mass in do	23200	23200	23200	23200	0
Mass in do	18902.93	18832.03	26419.78	21384.91	3560.308
Mass Balar	81.58599	81.36169	114.0409	92.32952	15.35252

Osmolality	50				
Barrier	PermeaPad (2 min pretreatment)				
Time	A	B	C	mean	SD
1800	3.346277	2.819065	2.615702	2.927014	0.307869
2700	4.882181	4.134232	3.325826	4.11408	0.635539
3600	6.781032	5.929752	5.06821	5.926331	0.699261
4500	8.123736	7.297134	6.25638	7.22575	0.764014
5400	9.469156	8.683058	7.587025	8.579746	0.771842
Flux (µg/cn	0.001721	0.001655	0.00143	0.001602	0.000124
Papp (10-6	0.593384	0.570532	0.493226	0.552381	0.042856
Mass in do	38619.41	38619.41	38619.41	38619.41	0
Mass in do	36571.7	38989.18	38801.07	38120.65	1097.963
Mass Balar	N/A	100.98	100.49	100.735	0.244962

Osmolality	300				
Barrier	PermeaPad (2 min pretreatment)				
Time	A	B	C	mean	SD
1800	1.022652	0.919154	1.44209	1.127965	0.226103
2700	1.754337	1.51386	2.249111	1.839103	0.306091
3600	3.313618	2.476483	3.020092	2.936731	0.346805
4500	4.030724	3.236028	3.630994	3.632582	0.324435
5400	5.713853	3.50439	4.854991	4.691078	0.909426
Flux (µg/cn	0.001295	0.000766	0.000912	0.000991	0.000223

Papp (10-6	0.446697	0.264086	0.314471	0.341751	0.077006
Mass in do	38619.41	38619.41	38619.41	38619.41	0
Mass in do	38705.4	40006.77	36971.93	38561.37	1243.148
Mass Balar	N/A	103.5924	95.73405	99.66322	3.929165

Acyclovir

Osmolality	50				
Barrier	PermeaPad				
Time	A	B	C	mean	SD
1800	8.642868	11.47176	6.787936	8.967521	1.925893
2700	12.50998	14.88181	9.139719	12.17717	2.355982
3600	15.74446	20.1892	12.03058	15.98808	3.335196
4500	20.03138	25.1589	16.98479	20.72502	3.372919
5400	22.68571	28.186	18.1283	23	4.11205
Flux (µg/cr	0.003956	0.004856	0.003392	0.004068	0.000603
Papp (10-6	3.956342	4.856175	3.391755	4.068091	0.603046
Mass in do	4.54E+09	4.54E+09	4.54E+09	4.54E+09	0
Mass in do		4.56E+09	5.42E+09	4.99E+09	4.28E+08
Mass Balar	N/A	100.4126	119.2611	109.8368	9.42426

Osmolality	150				
Barrier	PermeaPad				
Time	A	B	C	mean	SD
1800	7.185185	8.025222	6.862291	7.357566	0.490162
2700	11.69368	10.88174	11.45216	11.34253	0.340417
3600	13.46173	14.98806	14.79659	14.41546	0.678905
4500	17.36674	18.49908	19.26005	18.37529	0.777882
5400	20.05896	21.60277	22.03194	21.23122	0.84723
Flux (µg/cr	0.003491	0.003864	0.004239	0.003864	0.000305
Papp (10-6	3.49118	3.863604	4.238577	3.864453	0.305124
Mass in do	8000	8000	8000	8000	0
Mass in do	0	3.84E+09	4.29E+09	2.71E+09	1.93E+09
Mass Balar	N/A	48041659	53647048	50844353	2802694

Osmolality	300				
Barrier	PermeaPad				
Time	A	B	C	mean	SD
1800	3.336903	4.926821	1.992033	3.418586	1.199513
2700	5.594734	7.752007	5.797458	6.3814	0.972693
3600	7.607675	10.21861	8.030273	8.618854	1.144282
4500	10.2066	12.40671	8.57151	10.39494	1.571369
5400	13.23118	15.21101	9.912064	12.78475	2.186197
Flux (µg/cr	0.002711	0.002803	0.002068	0.002527	0.000327
Papp (10-6	2.711157	2.802566	2.068235	2.527319	0.32676
Mass in do	4.54E+09	4.54E+09	4.54E+09	4.54E+09	0
Mass in do	0	3.49E+09	4.23E+09	2.57E+09	1.84E+09
Mass Balar	N/A	76.75323	93.04274	84.89798	8.144755

Osmolality	450
Barrier	PermeaPad

Time	A	B	C	mean	SD
1800	5.538156	4.593473	7.89028	6.007303	1.386196
2700	8.744851	6.832923	11.86313	9.14697	2.073167
3600	11.91241	8.989881	15.94212	12.28147	2.850211
4500	15.44527	11.75468	20.33948	15.84648	3.516195
5400	18.79114	14.1526	26.16108	19.70161	4.944533
Flux (µg/cr	0.00369	0.002671	0.005002	0.003788	0.000954
Papp (10-6	3.663223	2.652018	4.966238	3.760493	0.947277
Mass in do	8057.6	8057.6	8057.6	8057.6	9.09E-13
Mass in do	-203.217	-203.217	-203.217	-203.217	0
Mass Balar	-2.28884	-2.34641	-2.19737	-2.27754	0.061365

Osmolality	900				
Barrier	PermeaPad				
Time	A	B	C	mean	SD
1800	9.604938	10.14885	7.271244	9.008343	1.248223
2700	11.61496	14.64508	11.30717	12.5224	1.506209
3600	15.68324	19.21094	16.90083	17.265	1.46302
4500	19.96433	23.33119	19.67644	20.99065	1.659177
5400	23.45828	29.05286	24.92064	25.81059	2.369086
Flux (µg/cr	0.004006	0.005166	0.004852	0.004675	0.00049
Papp (10-6	4.006227	5.166015	4.852006	4.67475	0.48979
Mass in do	4.54E+09	4.54E+09	4.54E+09	4.54E+09	0
Mass in do	0	4.52E+09	4.41E+09	2.98E+09	2.11E+09
Mass Balar	N/A	99.43186	97.07279	98.25233	1.179536

Osmolality	50				
Barrier	Support sheet				
Time	A	B	C	mean	SD
1800	63.70013	62.82106	59.6564	62.0592	1.736525
2700	101.4204	90.43197	90.46714	94.1065	5.171721
3600	128.5069	124.8675	123.883	125.7525	1.988718
4500	158.7581	143.972	151.7079	151.4793	6.038527
5400	193.0178	173.3793	179.1109	181.836	8.245701
Flux (µg/cr	0.035108	0.030517	0.03335	0.032992	0.001891
Papp (10-6	34.7605	30.21524	33.01977	32.66517	1.87246
Mass in do	8080	8080	8080	8080	0
Mass in do	6685.375	6685.375	5939.921	6436.89	351.4104
Mass Balar	85.12863	84.88557	75.73059	81.91493	4.374113

Osmolality	300				
Barrier	Support sheet				
Time	A	B	C	mean	SD
1800	66.33735	58.77732	68.27132	64.462	4.096477
2700	90.7836	80.53361	96.25144	89.18955	6.515023
3600	125.2192	122.1072	130.1595	125.8286	3.315472
4500	158.3888	137.2032	158.1251	151.239	9.925442
5400	184.9831	170.6542	197.2373	184.2915	10.86355
Flux (µg/cr	0.033877	0.031158	0.035534	0.033523	0.001804
Papp (10-6	33.54199	30.84964	35.18215	33.19126	1.78604

Mass in do	8080	8080	8080	8080	0
Mass in do	3717.624	6263.42	5616.422	5199.155	1080.387
Mass Balar	48.29959	79.62963	71.95123	66.62682	13.33304

Hydrocortisone

Osmolality	50				
Barrier	PermeaPad				
Time	A	B	C	mean	SD
1800	10.10755	9.764531	5.98928	8.620452	1.865782
2700	15.44229	14.40836	9.598403	13.14969	2.546364
3600	20.0793	18.27907	12.17616	16.84485	3.382074
4500	27.8856	26.86287	19.01755	24.58867	3.961443
5400	33.83946	30.79305	24.65979	29.7641	3.817559
Flux (µg/cn	0.006656	0.006057	0.005196	0.00597	0.0006
Papp (10-6	26.84482	23.75045	20.44708	23.68078	2.61233
Mass in do	1983.652	2040.159	2032.788	2018.866	25.08142
Mass in do	1951.714	1858.607	2144.563	1984.961	119.085
Mass Balar	100.0958	92.61043	106.7117	99.80599	5.760469

Osmolality	150				
Barrier	PermeaPad				
Time	A	B	C	mean	SD
1800	10.20582	6.69186	8.259263	8.385647	1.437348
2700	14.27285	10.0868	11.65126	12.00364	1.727015
3600	18.8902	13.45696	16.53355	16.29357	2.224591
4500	22.85404	18.16027	20.16734	20.39389	1.922909
5400	30.58664	21.97646	26.92977	26.49762	3.528345
Flux (µg/cn	0.005483	0.004294	0.005095	0.004957	0.000495
Papp (10-6	22.11088	16.83646	20.05219	19.66651	2.170476
Mass in do	1983.652	2040.159	2032.788	2018.866	25.08142
Mass in do	1983.652	1970.115	2536.081	2163.283	263.6662
Mass Balar	101.5419	97.64394	126.0835	108.4231	12.58876

Osmolality	300				
Barrier	PermeaPad				
Time	A	B	C	mean	SD
1800	7.749007	8.426432	4.311466	6.828968	1.801497
2700	12.70049	13.48161	7.604766	11.26229	2.605844
3600	17.77481	19.0076	13.10883	16.63041	2.540484
4500	23.53212	25.00935	15.83464	21.4587	4.022284
5400	27.29942	30.05461	21.32883	26.22762	3.642013
Flux (µg/cn	0.005548	0.006087	0.004696	0.005444	0.000573
Papp (10-6	22.3751	23.86921	18.48128	21.5752	2.271172
Mass in do	1983.652	2040.159	2032.788	2018.866	25.08142
Mass in do	1995.936	1947.813	2060.313	2001.354	46.08719
Mass Balar	101.9955	95.47392	101.3543	99.60788	2.934856

Osmolality	450				
Barrier	PermeaPad				
Time	A	B	C	mean	SD

1800	8.387778	5.601558	5.890585	6.62664	1.25089
2700	12.91177	9.283943	9.933966	10.70989	1.579417
3600	18.14824	13.77414	15.35908	15.76049	1.808137
4500	22.60836	17.28803	18.56848	19.48829	2.267308
5400	29.71201	22.07063	25.61219	25.79828	3.122356
Flux (µg/cn	0.005816	0.004549	0.005342	0.005236	0.000523
Papp (10-6	23.4562	17.83836	21.02322	20.77259	2.30031
Mass in do	1983.652	2040.159	2032.788	2018.866	25.08142
Mass in do	2059.813	2061.8	2184.211	2101.941	58.17902
Mass Balar	105.3373	102.1425	108.709	105.3963	2.681048

Osmolality	900				
Barrier	PermeaPad				
Time	A	B	C	mean	SD
1800	8.780868	9.764531	6.680145	8.408514	1.286428
2700	13.58985	13.21894	10.90118	12.56999	1.189706
3600	16.53166	19.10176	15.62555	17.08632	1.472354
4500	23.96452	24.85572	21.4109	23.41038	1.459909
5400	29.04867	29.38061	24.62524	27.68484	2.167703
Flux (µg/cn	0.005657	0.005652	0.005156	0.005488	0.000235
Papp (10-6	22.81326	22.16339	20.28955	21.7554	1.069927
Mass in do	1983.652	2040.159	2032.788	2018.866	25.08142
Mass in do	2099.122	2163.396	2142.085	2134.868	26.73132
Mass Balar	107.2855	107.4807	106.5881	107.1181	0.383133

Osmolality	50				
Barrier	Support sheet				
Time	A	B	C	mean	SD
1800	12.20301	12.46853	10.59354	11.75503	0.828417
2700	19.62025	19.17709	15.58837	18.12857	1.805281
3600	26.55433	24.96136	22.23609	24.58393	1.783001
4500	32.21227	31.66992	27.09449	30.32556	2.295416
5400	39.56648	35.78417	30.56235	35.30433	3.691548
Flux (µg/cn	0.00748	0.006569	0.005716	0.006588	0.00072
Papp (10-6	28.7524	25.25231	21.97197	25.32556	2.768581
Mass in do	2081.186	2081.186	2081.186	2081.186	0
Mass in do	2043.374	2043.609	2220.064	2102.349	83.23722
Mass Balar	100.0843	99.91388	108.1416	102.7132	3.839023

Osmolality	300				
Barrier	Support sheet				
Time	A	B	C	mean	SD
1800	8.73693	11.10311	9.911397	9.917145	0.965997
2700	13.33929	16.99241	15.25779	15.1965	1.492011
3600	19.20729	22.25677	20.98724	20.8171	1.250744
4500	23.4893	30.40953	27.30438	27.06773	2.830124
5400	28.71135	35.61086	30.81422	31.71214	2.887391
Flux (µg/cn	0.005567	0.006937	0.005984	0.006162	0.000574
Papp (10-6	21.39756	26.66541	23.00066	23.68788	2.204805
Mass in do	2081.186	2081.186	2081.186	2081.186	0

Mass in do	2093.79	2106.629	2026.804	2075.741	34.99834
Mass Balar	101.9852	102.9336	98.86759	101.2621	1.736903

Celecoxib

Osmolality	50				
Barrier	PermeaPad				
Time	A	B	C	mean	SD
7200	87.40203	82.88613	2.573804	57.62065	38.96764
14400	286.325	287.6999	45.85713	206.6273	113.6831
21600	505.7828	483.2143	92.83603	360.6111	189.5696
Flux (µg/cn	0.029054	0.027801	0.006268	0.021041	0.010458
Papp (10-6	23.51246	22.95531	7.384184	17.95065	7.475082
Mass in do	N/A	N/A	N/A	N/A	N/A
Mass in do	N/A	N/A	N/A	N/A	N/A
Mass Balar	N/A	N/A	N/A	N/A	N/A

Osmolality	150				
Barrier	PermeaPad				
Time	A	B	C	mean	SD
7200	98.93074	39.04647	13.80545	50.59422	35.69866
14400	224.8918	161.6276	62.02829	149.5159	67.03806
21600	340.985	302.6142	121.0601	254.8864	95.91733
Flux (µg/cn	0.016809	0.018303	0.007448	0.014187	0.004804
Papp (10-6	13.60314	15.1133	8.7743	12.49691	2.70352
Mass in do	N/A	N/A	N/A	N/A	N/A
Mass in do	N/A	N/A	N/A	N/A	N/A
Mass Balar	N/A	N/A	N/A	N/A	N/A

Osmolality	300				
Barrier	PermeaPad				
Time	A	B	C	mean	SD
7200	65.25777	28.78098	65.49519	53.17798	17.25155
14400	205.7154	114.2253	195.6487	171.8631	40.96278
21600	382.7224	187.206	320.3071	296.7452	81.53949
Flux (µg/cn	0.022046	0.011002	0.017695	0.016914	0.004543
Papp (10-6	17.8411	9.084287	20.84569	15.92369	4.98932
Mass in do	N/A	N/A	N/A	N/A	N/A
Mass in do	N/A	N/A	N/A	N/A	N/A
Mass Balar	N/A	N/A	N/A	N/A	N/A

Osmolality	450				
Barrier	PermeaPad				
Time	A	B	C	mean	SD
7200	8.812744	82.52382	43.51497	44.95051	30.10954

14400	73.60888	231.4329	198.9578	167.9999	68.04853
21600	157.3361	375.6803	345.5481	292.8548	96.6126
Flux (µg/cn	0.010314	0.020358	0.020975	0.017216	0.004887
Papp (10-6	8.346819	16.80996	24.70877	16.62185	6.681061
Mass in do	N/A	N/A	N/A	N/A	N/A
Mass in do	N/A	N/A	N/A	N/A	N/A
Mass Balar	N/A	N/A	N/A	N/A	N/A

Osmolality	900				
Barrier	PermeaPad				
Time	A	B	C	mean	SD
7200	69.53823	93.39315	101.9679	88.29975	13.72048
14400	221.8955	171.0477	230.7446	207.8959	26.30486
21600	367.6437	269.8734	428.1068	355.208	65.19426
Flux (µg/cn	0.020702	0.012256	0.022649	0.018535	0.004511
Papp (10-6	16.75314	10.11959	26.68081	17.85118	6.805526
Mass in do	N/A	N/A	N/A	N/A	N/A
Mass in do	N/A	N/A	N/A	N/A	N/A
Mass Balar	N/A	N/A	N/A	N/A	N/A

Osmolality	50				
Barrier	Support sheet				
Time	A	B	C	mean	SD
7200	254.3392	251.5814	251.5814	252.5007	1.300048
14400	544.4055	582.9276	599.9453	575.7595	23.23364
21600	906.2693	915.4307	845.335	889.0117	31.10971
Flux (µg/cn	0.045273	0.046101	0.041233	0.044202	0.002127
Papp (10-6	47.45724	48.32489	43.22228	46.3348	2.229209
Mass in do	N/A	N/A	N/A	N/A	N/A
Mass in do	N/A	N/A	N/A	N/A	N/A
Mass Balar	N/A	N/A	N/A	N/A	N/A

Osmolality	300				
Barrier	Support sheet				
Time	A	B	C	mean	SD
7200	215.5952	271.6933	262.2763	249.8549	24.52844
14400	536.6164	579.2079	600.2615	572.0286	26.47427
21600	895.7964	905.8254	850.2991	883.9736	24.16094
Flux (µg/cn	0.047236	0.044037	0.040835	0.044036	0.002613
Papp (10-6	49.51523	46.16163	42.8051	46.16065	2.739397
Mass in do	N/A	N/A	N/A	N/A	N/A
Mass in do	N/A	N/A	N/A	N/A	N/A

Mass Balar	N/A	N/A	N/A	N/A	N/A
